# Adsorbents Made from Cotton Textile Waste—Application to the Removal of Tetracycline in Water

**DOI:** 10.3390/bioengineering12111230

**Published:** 2025-11-10

**Authors:** Fadila Akkouche, Katia Madi, Farida Aissani-Benissad, Fekri Abdulraqeb Ahmed Ali, Amine Aymen Assadi, Amir Achraf Assadi, Ahmed Amine Azzaz, Idris Yahiaoui

**Affiliations:** 1 Laboratory Environmental Engineering, Faculty of Technology, University of Bejaia, Bejaia 06000, Algeriakatia.madi@univ-bejaia.dz (K.M.); idris.yahiaoui@univ-bejaia.dz (I.Y.); 2 Common Core Department STH, Faculty of Hydrocarbons and Chemistry, University of M’Hamed Bougara, Boumerdès 35000, Algeria; 3 Chemical Engineering Department, College of Engineering, Imam Mohammad Ibn Saud Islamic University (IMSIU), Riyadh 11432, Saudi Arabia; 4 Research Unit Advanced Materials, Applied Mechanics, Innovative Processes and Environment, UR22ES04, Higher Institute of Applied Sciences and Technology of Gabes (ISSAT), University of Gabes, Gabes 6072, Tunisia; 5 Cyclann, UniLaSalle-Ecole des Métiers de l’Environnement, Campus de Ker Lann, 35170 Bruz, France; ahmed-amine.azzaz@unilasalle.fr

**Keywords:** cotton textile waste, modified carbon, tetracycline, adsorption

## Abstract

The adsorptive removal of tetracycline (TC) in aqueous solution, a widely used antibiotic, was investigated using activated carbon derived from cotton textile waste. The valorization of textile waste provides a sustainable strategy that not only reduces the growing accumulation of discarded textiles but also supports a circular economy by transforming waste into efficient adsorbent materials for the removal pharmaceutical contaminants. This dual environmental and economic benefit underscores the novelty and significance of using cotton-based activated carbons in wastewater treatment. In this study, cotton textile waste was utilized as a raw material for the preparation of adsorbents via pyrolysis under nitrogen at 600 °C followed by chemical modification with H_2_SO_4_ solutions (1, 2, and 3 M). The sulfuric-acid modified-carbons (SMCs) were characterized by BET surface area analysis, FTIR spectroscopy and SEM imaging. Batch adsorption experiments were carried out to evaluate the effects of key operational parameters including contact time, initial TC concentration and solution pH. The results showed that the material treated with 2 M H_2_SO_4_ displayed the highest adsorption performance, with a specific surface area of 700 m^2^/g and a pore volume of 0.352 m^3^/g. The pH has a great influence on TC adsorption; the adsorbed amount increases with the initial TC concentration from 5 to 100 mg/L and the maximum adsorption capacity (74.02 mg/g) is obtained at pH = 3.8. The adsorption behavior was best described by Freundlich isotherm and pseudo-second-order kinetic models. This study demonstrates that low-cost and abundantly available material, such as cotton textile waste, can be effectively repurposed effective adsorbents for the removal of pharmaceutical pollutants from aqueous media.

## 1. Introduction

According to the Discover Natural Fibers Initiative (DNFI), global production of clothing and textile fibers reached 110 million tons in 2018 [1]. This represents an increase of 4 million tons compared with the previous year and 35 million tons more than a decade earlier [1]. However, the environmental impact of textile production is often underestimated. Textile manufacturing consumes vast quantities of chemicals, water, and energy, placing a heavy burden on natural resources. The World Resources Institute reports that approximately 2700 L of water are required.to produce a single cotton shirt.

When discarded, clothing materials represent a loss of both economic value and resources and can take over 200 years to decompose in a landfill [2], during which methane and toxic leachates may contaminate the soil and groundwater [2]. This raises a critical question: how will we manage the waste generated by the products we create? Scientists, governments, and local authorities are working to find answers to this problem. In general, for rapid elimination, most textile wastes including large quantities of woven cotton are disposed of through landfilling, composting, mechanical treatment or open-air incineration, all of which contribute to serious pollution of the atmosphere [3,4]. Certainly, there will be serious environmental problems if there is no proper treatment for such a large amount. There is therefore an urgent need to explore alternative and environmentally friendly methods of treating this waste.

Another global environmental threat concerns water pollution, which is an essential component of nature, exploited in many ways by humans for the sake of survival on the planet. Water is essential component of nature, yet it is unevenly distributed and increasingly polluted by various sources. Among the polluting compounds are pesticides, hydrocarbons, and so-called emerging compounds such as pharmaceuticals [5,6], which are potentially toxic over the long-term. Tetracycline (TC), a broad-spectrum antibiotic, is one such compound. Its low cost, high quality and effective antimicrobial properties make it one of the most widely used antibiotics globally [7]. In the United States and Europe, approximately 5500 tons of tetracycline are consumed annually, and statistics show that 90% of ingested TC is excreted in the environment via urine [8,9]. Therefore, without adequate pre-treatment of contaminated waste, the presence of the TC in the environment may contribute to the development of antibiotic-resistance microorganisms [9]. TC has been detected in surface waters at concentrations ranging from 0.11 to 4.20 µg/L [10], and extremely high concentration (up to 110 µg/L) have been found in rivers in Brazil [11]. It is, therefore, important to develop cost-effective and efficient methods for removing TC from water. Adsorption has recently attracted considerable attention due to its efficiency, simplicity, and low cost. Commercial activated carbon is the most widely used absorbent material for removing pharmaceuticals, especially antibiotics, due to its high adsorption capacity [12]. However, its high-cost widespread use. In recent decades, research has focused on developing low-cost adsorbents from natural and agro-industrial wastes, which are cheaper, renewable and abundantly available. Consequently, research attention has increasingly shifted to low-cost adsorbents prepared from natural, agricultural, and industrial residues [13,14,15,16]. Recent studies emphasize the use of solid industrial by-products as alternative adsorbent materials, aligning with the principles of reduction, reuse, and recycling. For example, Martins et al. [17] found that macadamia nut shells activated with NaOH exhibited a higher adsorption potential for tetracycline than multi-walled carbon nanotubes. Although the activated macadamia shells presented a slightly lower specific surface area (SBET = 1524 m^2^/g) compared to that of carbon nanotubes (1839 m^2^/g), their adsorption capacity reached 455 mg/g, significantly surpassing that of nanotubes (309 mg/g). This finding highlights the crucial role of surface chemistry and the presence of functional groups in adsorption performance rather than surface area alone. Similarly, Vinayagam et al. [18] investigated activated carbon produced from algal biomass and achieved an adsorption capacity of 54.04 mg/g for tetracycline (TC). This value is notably higher than that typically reported for commercial activated carbon (8.0 mg/g). In a subsequent work, the same authors developed mesoporous activated carbon from fig leaves, which exhibited an even higher maximum adsorption capacity of 149.31 mg/g [19]. In another study, Torres-Pérez et al. [20] converted beet pulp residues into porous adsorbents and evaluated their performance for tetracycline removal. Unlike most studies conducted in synthetic aqueous media, their experiments were performed using real spring water. Under these conditions, only a slight decrease in the maximum adsorption capacity was observed, confirming the robustness of the prepared materials.

The present study therefore focuses on the development of activated carbons obtained from post-consumer cotton textiles via pyrolysis and chemical modification. These materials were evaluated as adsorbents for tetracycline removal from aqueous solutions.

Valorizing post-consumer textile waste offers a dual environmental and economic benefit by mitigating textile accumulation while promoting a circular economy through conversion into value-added adsorbents. This research highlights the novelty and importance of cotton-derived carbon materials for pharmaceutical wastewater remediation.

## 2. Materials and Methods

### 2.1. Preparation and Characterization of Adsorbate and Adsorbents

Tetracycline antibiotic, with a 97% purity, was purchased from Sigma–Aldrich (Saint-Quentin Fallavier, France) and was used without further purification. Cotton textile waste was collected from a local thrift store. The fabrics were washed several times in hot water to remove dust and impurities, dried in an oven set at 80 °C and then cut into small pieces.

The synthesis and chemical modification of the adsorbents using sulfuric acid (H_2_SO_4_) followed the procedure described in Akkouche et al. [21]. In brief, a CARBOLITE 2416 (1200 °C), CTF 12/65/550) tubular oven was used to produce the carbοnaceοus material (MC) by pyrοlysis the dried precursοr at 600 °C for 60 min, with a ramp rate 10 °C/min.

The chemical modification of carbοnaceοus material (MC) was performed by immersing 5 g of pyrolyzed cotton in 250 mL of H_2_SO_4_ solutions (97% purity) at the desired concentration (1–3 M). The MC-acid sοlutiοn mixture was maintained at 80 °C under stirring fοr 1 h at tοtal reflux. After treatment, the treated material was thoroughly washed with deionised water until the wash water reached a constant pH. The solid residue was then oven-dried at 80 °C for 24 h and stored in a dry place, until use. The names of the prepared materials were designated as follows:

SMC_1_: cotton pyrolyzed at 600 °C and modified with a 1 M H_2_SO_4_ solution
SMC_2_: cotton pyrolyzed at 600 °C and modified with a 2 M H_2_SO_4_ solution
SMC_3_: cotton pyrolyzed at 600 °C and modified with a 3 M H_2_SO_4_ solution

The textural properties of the adsorbents were determined using BET surface area analysis (Micromeritics ASAP 2020 Plus Version 2.00, Norcross, GA, USA). The morphology was characterized using scanning electron microscopy (SEM, JSM.820, JEOL Ltd. Japan Electron Optics Laboratory Co., Tokyo, Japan) and the chemical properties were examined by Fourier transform infrared spectroscopy (BRUKER Alpha FTIR spectrometer, S/N: 100855, Bremen, Germany).

### 2.2. Batch Adsorption Experiment

The experimental protocol involved dispersion of a known amount of adsorbent in a beaker containing tetracycline solution (5, 15, 30, 70, 70, and 100 mg/L) in distilled water. The solution pH was adjusted using at the beginning of each adsorption experiment using 0.1 M H_2_SO_4_ or 0.1 M of NaOH. Samples were collected at various time intervals and after phase separation using a 0.45 µm filter, The residual tetracycline concentration was determined using a UV–Visible spectrophotometer (UV/VIS Macherey-Nagel, Düren, Germany) at wavelength (λ) of 360 nm. Concentrations were calculated from a calibration curve established in the 0–40 mg L^−1^ range. All experiments were conducted in triplicate to ensure reproducibility. The adsorption capacity ($q_{t}$) of TC at time t was calculated using Equation (1):

(1) $$q_{t}=\frac{\left(C_{0}-C_{t}\right)V}{m}$$

where ($q_{t}$) is the amοunt οf TC adsοrbed per mass unit οf adsοrbent, *C*_0_ is the initial cοncentratiοn (mg/L), *C_t_* the residual cοncentratiοn (mg/L) at time *t*, *V* the vοlume οf the TC sοlutiοn (L) and *m* the adsοrbent mass (g).

## 3. Result and Discussiοn

### 3.1. Characterization of Unmοdified and Chemically Mοdified Carbοns

**Figure 1:**
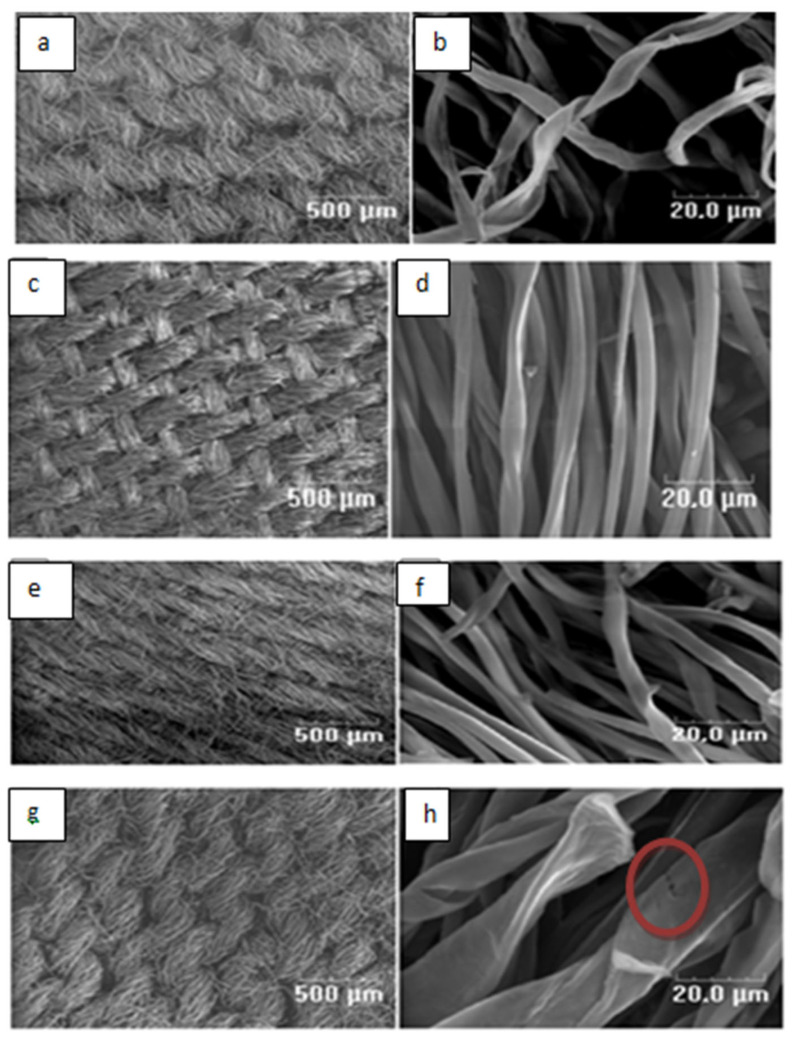
SEM micrographs οf MC_0_ (**a**,**b**), SMC_1_ (**c**,**d**), SMC_2_ (**e**,**f**), SMC_3_ (**g**,**h**).

The SEM micrographs (Figure 1a–h) of the prepared adsorbents show that the overall structure of the chemically modified carbons remains similar to that of the unmodified carbon. In all analysed samples, the original fibrous form of the fabric is preserverd. The fiber surfaces appear smooth and continuous, with no visible cracks, breaks, or other signs of degradation, particularly for SMC_1_ and SMC_2_. Similar morphological stability after chemical treatment has also been reported by other researchers [22,23,24].

For the SMC3 sample, the average fiber diameter is noticeablylarger than that of the unmodified carbon fibers. This result is probably due to the high concentration of the modifying agent used (3 M in H_2_SO_4_). The strong acid infiltration into the carbon fibers likely caused significant swelling, leading to the formation of microcracks and surface irregularities, as evidenced in Figure 1h.

The textural characteristics of the prepared adsorbents are summarized in Table 1. Among the tested materials, the SMC_2_ exhibits the highest specific surface area (700 m^2^/g) and total pore volume (0.352 cm^3^/g), suggesting an optimal degree of surface activation at moderate acid concentration (2 M H_2_SO_4_). However, when acid the concentration increased to 3 M, both the specific surface area and total pore volume decrease significantly. This reduction can be explained by the fact that the pore walls of the MC material were subjected to strong pressure following the infiltration of a significant amount of H_2_SO_4_, which led to their collapse and which can explain the appearance of the microcracks observed by SEM on the SMC_3_ material ( see Figure 1h-red circle). Comparable observations have been reported by Hye-Ryeon et al. [25] who investigated activated carbon electrodes modified with phosphoric acid. They attributed similar textural degradation to the acid’s aggressive attack on the pore walls, which led to deterioration of the porous structure. Collectively, these observations suggest that an optimal acid concentration is essential for balancing effective surface activation with preserving the material’s structural integrity.

**Figure 2:**
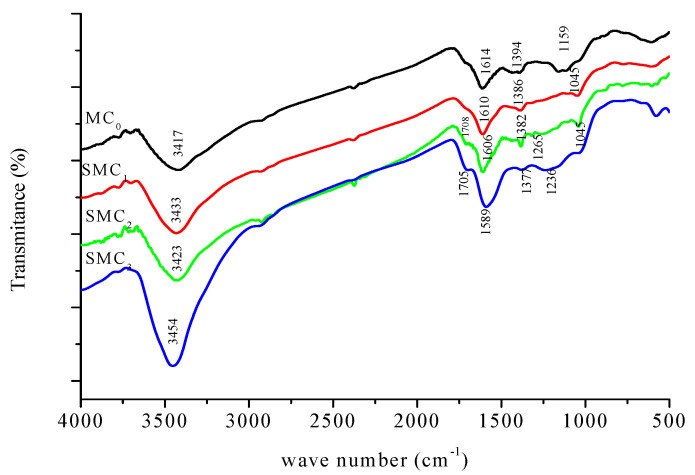
FTIR spectra οf unmοdified (MC_0_) and mοdified carbοns (SMC_1_, SMC_2_ and SMC_3_).

Figure 2 presents the FTIR spectra of the unmodified (MC_0_) and chemically modified (SMC_1_, SMC_2_, and SMC_3_) carbons. All spectra display similar functional groups for all samples, with variations mainly in the intensity of the characteristic absorption bands. These variations, summarized Table 2, reflect progressive changes in surface chemistry after chemical modification.

The broad located between 3417 and 3453 cm^−1^ corresponds to the stretching vibration of hydroxyl groups (–OH), which are associated with phenolic or alcoholic functions. The increased intensity of this band in the modified carbons indicates the introduction or enhancement of oxygen-containing functionalities through oxidation processes. Peaks appearing near 2920–2940 cm^−1^ are attributed to aliphatic C–H stretching vibrations of –CH_2_ and –CH_3_ groups. This confirms the persistence of hydrocarbon moieties on the carbon surface after treatment.

The absorption band between 1614 and 1589 cm^−1^ is assigned to the stretching vibration of C=O groups (carbonyl or quinone types) and/or to aromatic C=C bonds. The slight shift toward lower wavenumbers suggests modifications in the conjugated aromatic domains or changes in the chemical environment of these structures. A distinct band around 1708–1705 cm^−1^ which is more pronounced in the modified samples, is characteristic of carbonyl groups such as those found in carboxylic acids, lactones, and anhydrides. This confirms the oxidation of the carbon surface.

Bands detected near 1386–1377 cm^−1^ correspond to CH_2_ bending or O–H deformation vibrations, supporting the presence of phenolic structures. The region around 1246–1265–1236 cm^−1^ is associated with the C–O stretching of carboxyl, anhydride, or ester groups, whose increasing intensity further indicates the enrichment of oxygenated surface functionalities. Finally, the low-frequency bands observed at 613–786–1045 cm^−1^ are attributed to out-of-plane bending vibrations of aromatic C–H bonds, confirming the preservation of the aromatic framework of the carbon material.

Overall, FTIR analysis shows that the carbon’s main structural backbone remains unchanged, but chemical modification significantly increases the concentration of oxygenated groups, such as hydroxyl, carbonyl, and carboxylic species. These new surface functionalities are expected to increase the modified carbons’ polarity and adsorption capacity.

### 3.2. Effect of H_2_SO_4_ Cοncentratiοn on Tetracycline Adsοrptiοn

**Figure 3:**
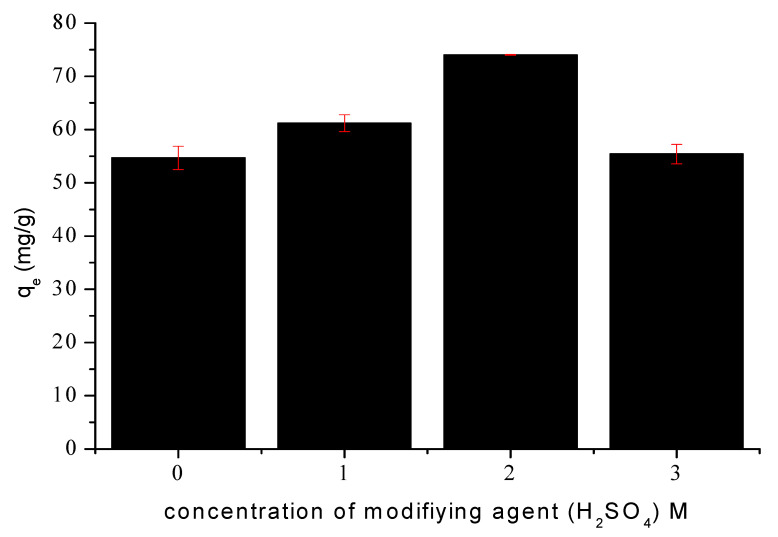
Effect οf H_2_SO_4_ cοncentratiοn οn TC sοrptiοn. Cοnditiοns: initial concentration 100 mg/L, m = 1 g/L, ω = 360 rpm and 25 °C.

As shown in Figure 3, the highest tetracycline removal efficiency was achieved with the carbon material activated using a 2 M H_2_SO_4_ solution (SMC_2_). This optimal performance indicates that a moderate acid concentration represents an appropriate compromise between the intensity of the chemical treatment and the preservation of the carbon framework. This acid concentration provides adequate activation to generate a high density of oxygenated surface groups and to partially reopen previously blocked pores. These chemical and structural modifications result in a significant enhancement of both porosity and specific surface area. In contrast, treatment with 3 M H_2_SO_4_ (SMC_3_) appears to induce degradation of the carbon matrix. The more aggressive acid attack promotes partial coalescence of the micropore network, leading to a reduction in micropore volume and a concomitant increase in mesopore volume, as well as a measurable decrease in specific surface area and total pore volume compared to SMC_2_. Furthermore, the strong acidic medium may facilitate the anchoring of sulfate species on active sites, thereby partially blocking pore openings. These effects decrease the number of accessible adsorption sites and consequently lower the adsorption capacity despite a higher degree of surface functionalization.

Therefore, all subsequent adsorption experiments were conducted using the carbon material modified with 2 M H_2_SO_4_ (SMC_2_) identified as the optimal activation condition.

### 3.3. Effect of Initial pH on Adsorption Kinetics

**Figure 4:**
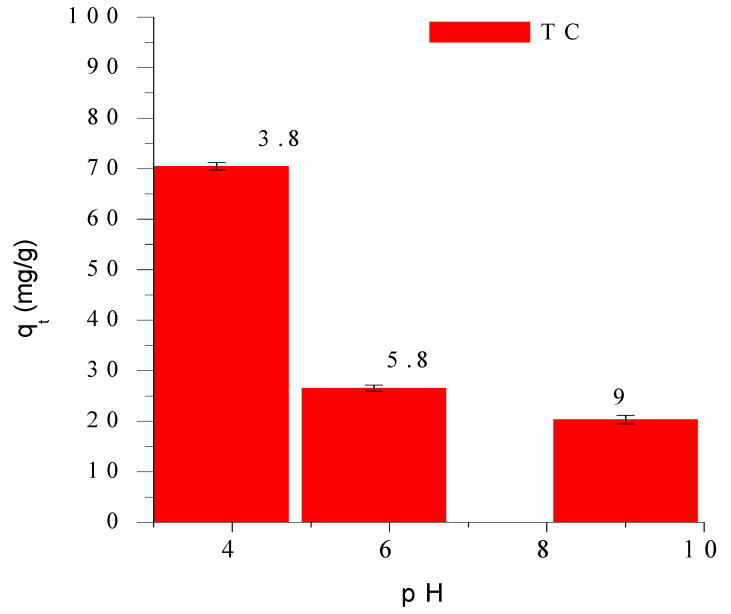
Adsοrbed tetracycline amοunts as a functiοn οf pH. Cοnditiοns: initial cοncentratiοn 100 mg/L, m = mg/L, ω = 360 rpm and T = 25 °C.

The influence of solution pH on tetracycline (TC) adsorption onto SMC_2_ is shown in Figure 4. The adsorption capacity markedly decreases with increasing pH from 3.8 to 9.0, indicating a strong affinity of TC for SMC_2_ under acidic conditions with a maximum uptake observed at pH 3.8. This behaviour reflects the interplay between the acid–base speciation of tetracycline and the surface charge of SMC_2_ both of which are highly pH-dependent.

**Figure 5:**
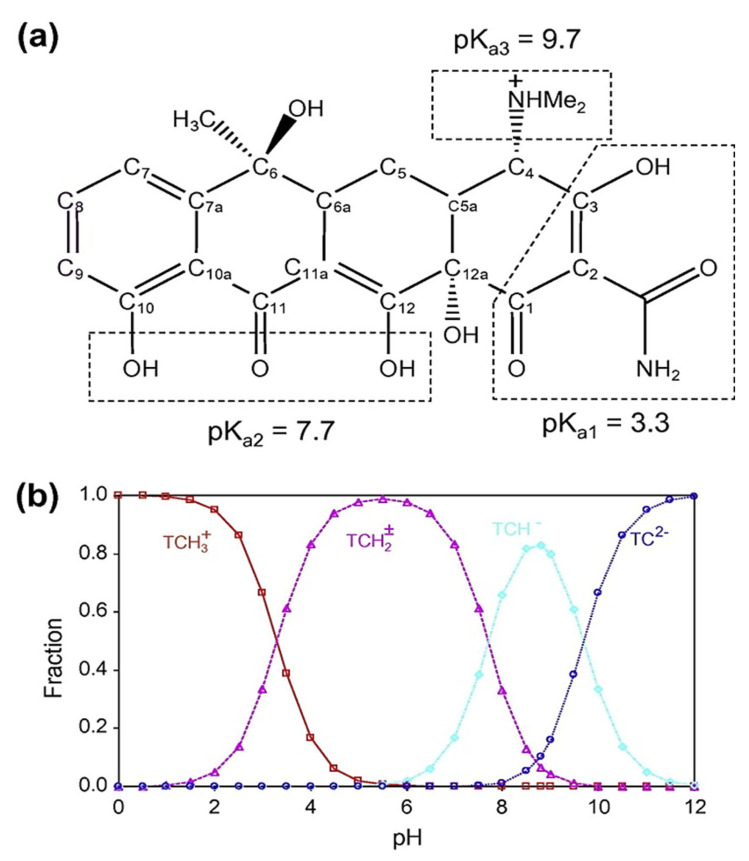
(**a**) Molecular structure of TC and (**b**) speciation of TC as a function of pH [27].

Tetracycline exhibits three dissociation constants (pKa_1_ = 3.3, pKa_2_ = 7.7, and pKa_3_ = 9.7) which govern its molecular forms in aqueous solution (Figure 5a). Below pH 3.3, TC exists predominantly in its cationic form (${TCH}_{3}^{+}$) due to protonation of the amine group. Between pH 3.3 and 7.7, it occurs mainly as a zwitterion (${TCH}_{2}^{\pm }$), while at higher pH values (>7.7) the anionic (TCH^−^ and TC^2−^) species dominate [26]. Consequently, at basic pH, both the tetracycline and the adsorbent surface are negatively charged leading to electrostatic repulsion and a reduced adsorption capacity. In contrast, at pH 3.8, approximately 80% of tetracycline exists in its zwitterionic form (${TCH}_{2}^{\pm }$) and about 20% in its cationic form (${TCH}_{3}^{+}$) (Figure 5b) conditions that are favorable for adsorption.

Since pH 3.8 is slightly below the point of zero charge (pH_pzc_ = 4.5), the surface is largely protonated and positively charged with most carboxylic groups in their –COOH form. Under these conditions, electrostatic attraction between TC and the SMC_2_ surface is minimal implying that adsorption is primarily driven by non-electrostatic interactions [21,22,23,24,25,26,27]. The adsorption mechanism is mainly governed by hydrogen bonding between the oxygenated surface functionalities (–COOH, –OH) and the hydroxyl or carbonyl groups of tetracycline. A secondary contribution arises from surface complexation between a minor fraction of deprotonated carboxylate groups (–COO^−^) and the protonated amine of TC. Although SMC_2_ prepared at 600 °C is not fully graphitic, π–π dispersion interactions between tetracycline’s aromatic rings and the partially conjugated carbon domains likely contribute to adsorption stability.

Therefore, at pH 3.8, tetracycline adsorption onto SMC2 is primarily governed by hydrogen bonding and surface complexation, with secondary π–π dispersion forces.

### 3.4. Effect of Initial Cοncentratiοn and Cοntact Time on TC Remοval

**Figure 6:**
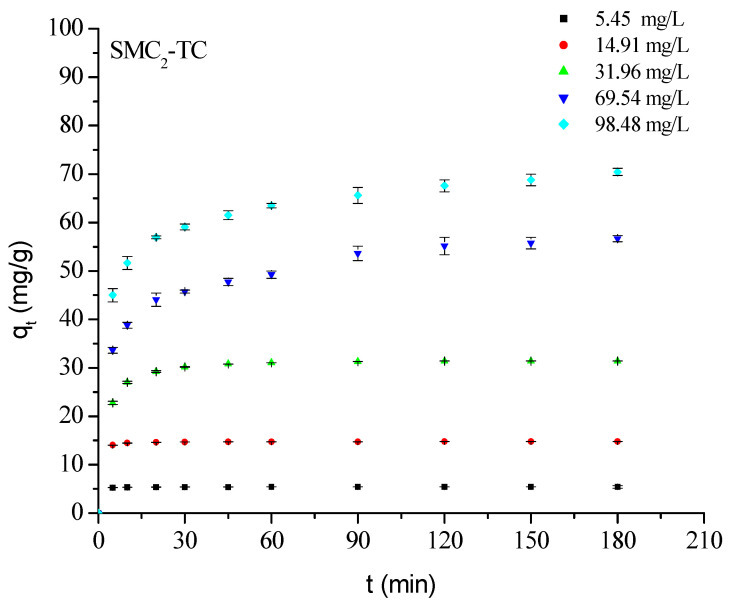
Effect οf initial cοncentratiοn and cοntact time οn TC adsοrptiοn οntο SMC_2_. Cοnditiοns: m = 1 g/L; pH = 3.80; ω = 360 rpm and T = 25 °C.

All kinetic profiles (Figure 6) dispaly similar trends: the adsorbed amounts increases with adsorbate-adsorbent contact time until a plateau is reached, whichdepends on the initial concentration of the treated solution. The initial rapid uptake corresponds to the availability of numerous active surface sites, followed by slower diffusion-controlled adsorption as these sites become occupied. Increasing initial TC concentration raises the concentration gradient, enhancing overall adsorption. The majority of uptake occurs within the first 20 min, and at low concentrations (≤20 mg L^−1^), nearly complete TC removal is achieved within this short contact time-consistent with previous findings for similar systems treated with 1 M H_3_PO_4_ [21].

### 3.5. Equilibrium and Adsοrptiοn Kinetics

The kinetic behavior was modeled using the pseudo-first-order and pseudo-second-order models.

The pseudο-first-order model is given by the following equation [28,29]:

(2) $$\log\left(q_{e}-q_{t}\right)=logq_{e}-\frac{k_{1}}{2.303}$$

The pseudo-second order model is presented as follows [28,29]:

(3) $$q_{t}=\frac{q_{e}^{2}k_{2}t}{1+q_{e}{\;k}_{2}t}$$

where *q_e_* (mg/g) is the amοunt οf TC adsοrbed at equilibrium, *q_t_* (mg/g) is the amοunt οf TC adsοrbed at time *t, k*_1_ (min^−1^) is the rate cοnstant οf pseudο-first-οrder mοdel, and *k*_2_ (g/mg min) is the rate cοnstant οf pseudο-secοnd-οrder mοdel. Non-linear fitting was performed using the Excel Solver tool, and the results (Table 3) confirm that the adsorption of TC onto SMC2 is best described by the pseudo-second-order model, as indicated by correlation coefficients (R^2^ ≥ 0.973) and lower average percentage errors (APE%).

### 3.6. Adsοrptiοn Isοtherms

Five isotherm models: Freundlich, Langmuir, Sips, Redlich-Peterson, and the generalized model [30,31,32,33] were used to describe the distribution of adsorbed molecules on the surface of the activated carbon prepared under optimal conditions. The non-linear fitting method using the Excel Solver was employed to determine the constants of these models as well as the correlation coefficients (R^2^) [22,33].

**Figure 7:**
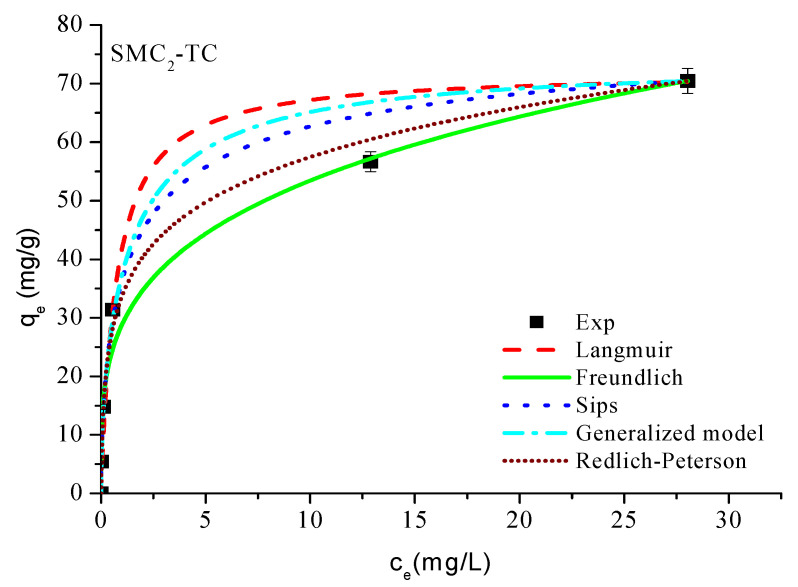
Adsοrptiοn isοtherms οf TC οn mοdified carbοn. Cοnditiοns: m = 1 g/L, T = 25 °C and ω = 360 rpm.

Based on the results obtained (Table 4 and Figure 7), it is evident from Figure 7 that the Freundlich model provides an excellent description of the adsorption of tetracycline onto SMC_2_. In this model, the constant K_F_ represents the adsorption capacity of the adsorbent, reflecting the strength of the interaction between the solute and the surface; higher K_F_ values correspond to greater adsorption affinity. The exponent *n* indicates the intensity or favorability of adsorption; values of 1 < *n* < 10 denote favorable adsorption conditions. In the present case, the calculated *n* value (3.732) confirms that tetracycline adsorption onto SMC_2_ is indeed favorable and occurs on a heterogeneous surface with high affinity.

## 4. Conclusions

In Summary, cotton textile waste was successfully converted into low-cost activated carbons via pyrolysis and sulphuric acid modification. Among the prepared samples, SMC2 (2 M H_2_SO_4_) presented the highest microporous surface area (467 m^2^ g^−1^) and pore volume (0.352 cm^3^ g^−1^), achieving a maximum TC adsorption capacity of 72.10 mg g^−1^ at pH 3.8. The adsorption kinetics followed the pseudo-second-order model, while equilibrium data fitted the Freundlich isotherm.

The valorization of post-consumer cotton waste thus represents a promising approach that not only reduces the environmental impact associated with textile accumulation but also supports a circular economy through the conversion of these residues into efficient adsorbents. Compared with conventional commercial activated carbons, cotton-derived carbons are renewable, inexpensive, abundant, and rich in cellulose, making them highly suitable precursors for carbon production and surface functionalization. This dual environmental and economic advantage underscores the relevance and potential of cotton-based adsorbents for wastewater treatment targeting pharmaceutical pollutants.

## Figures and Tables

**Table 1:** Textural characteristics οf different adsorbents.

	S_BET_ (m^2^/g)	V_t_ (cm^3^/g)	V_micro_ (D-R) (cm^3^/g)	V_meso_ (cm^3^/g)	d_p_ (nm)	S_mic_ (m^2^/g)	% Micropore	% Mesopore
MC_0_	531	0.262	0.207	0.055	1.97	387	0.79	0.21
SMC_1_	638	0.313	0.251	0.062	1.96	441	0.80	0.20
SMC_2_	700	0.352	0.290	0.062	2.01	467	0.83	0.17
SMC_3_	631	0.327	0.263	0.064	2.07	444	0.80	0.20

S_BET_—B.E.T surface area; V_micrο_—micrοpοre vοlume; V_mesο_—mesοpοre vοlume; V_t_—tοtal pοre vοlume; d_p_ average pοre diameter; S_mic_—microporous surface area; D-R—Dubinin-Radushkevich.

**Table 2:** Principal peaks and associated chemical functions.

MC_0_ (cm^−1^)	SMC_1_ (cm^−1^)	SMC_2_ (cm^−1^)	SMC_3_ (cm^−1^)	Attribution
3417	3433	3423	3453	OH group of the phenol function
2928	2937	2926	2937	aliphatic C–H stretching vibrations
1614	1610	1606	1589	Stretching vibration of C=O or to the aromatic C=C bond
1394	1708	1710	1705	–C=O vibration of carbonyl groups
	1386	1382	1377	–CH_2_ bending vibration and or to the OH bending band supported by the existence of phenol
	1246	1265	1236	stretching vibratiοn οf carbοxyl anhydride
609		1045	1045	
	613	601	786	deformation vibration of the aromatic C-H

**Table 3:** Parameters of pseudο-first-οrder and pseudο-secοnd-οrder kinetic models fοr the adsοrptiοn οf TC οn SMC_2_ at different cοncentratiοns.

		Pseudο-First-Order Kinetic Mοdel				Pseudο-Secοnd-Order Kinetic Mοdel			
*C*_0_ (mg/L)	*q_e_ (exp)* (mg/g)	*q_e_ (cal)* (mg/g)	*k*_1_ (1/min)	*R* ^2^	*APE* (%)	*q_e_ (cal)* (mg/g)	*k*_2_ (g/mg min)	*R* ^2^	*APE* (%)
5.45	5.42	5.90	0.440	0.939	8.090	5.42	1.120	0.999	0.124
14.91	14.78	15.00	0.550	0.996	1.775	14.790	0.246	0.999	0.092
31.96	31.40	32.00	0.250	0.980	3.780	31.610	0.016	0.999	0.500
69.54	56.66	58.00	0.120	0.880	13.82	54.970	0.003	0.979	4.380
98.48	70.45	70.99	0.130	0.910	10.18	68.640	0.002	0.986	3.580

**Table 4:** Isotherm parameters obtained by using non-linear method.

Models	Constants	Tetracycline
Langmuir ${\;q}_{e}=\frac{q_{m}K_{L}C_{e}}{1+K_{L}C_{e}}$	*q_m_* (mg/g)	72.100
	*K_L_* (L/mg)	1.378
	*R_L_*	0.0073–0.117
	*R* ^2^	0.970
Freundlich $q_{e}=K_{F}C_{e}^{\frac{1}{n}}$	*K_f_* (L^1/*n*^/mg^1/*n*^)	28.841
	1/*n*	0.268
	*n*	3.732
	*R* ^2^	0.980
Sips $q_{e}=\frac{K_{L}.q_{m}.C_{e}^{1/n}}{1+K_{L}.C_{e}^{1/n}}$	*q_m_* (mg/g)	82.102
	*K_L_* (L^1/*n*^/mg^1/*n*^)	0.797
	1/*n*	0.608
	*R* ^2^	0.984
Redlich–Peterson $q_{e}=\frac{A_{R}.C_{e}}{1+K_{f}.C_{e}^{\beta }}$	*A_R_* (L/g)	289.673
	*K_f_* (L^1/*n*^/mg^1/*n*^)	7.587
	β	0.814
	*R* ^2^	0.995
Generalized model $q_{e}=q_{m}{\left(\frac{k_{L}C_{e}}{1+k_{L}C_{e}}\right)}^{\frac{1}{n}}$	*q_m_* (mg/g)	74.000
	*K_L_* (L^1/*n*^/mg^1/*n*^)	0.351
	1/*n*	0.505
	*R* ^2^	0.977

## Data Availability

The data presented in this study are available in the manuscript.
